# A Compend of Surgery

**Published:** 1890-12

**Authors:** 


A COMPEND OF SURGERY. For students and physicians. By ORVILLE HORWITZ, B. S., M. D., Demonstrator of Anatomy in Jefferson Medical College; Chief of the Out-door Surgical Department of Jefferson Medical College Hospital, and late Resident Surgeon of the Pennsylvania Hospital, Philadelphia. Quiz Compends No. 9. Third edition; thoroughly revised, enlarged and improved, with 91 illustrations. Philadelphia: P. Blakiston, Son & Co. 1887. Buffalo: Peter Paul & Bro.

There is a large amount of surgical knowledge compressed into a small space in this little book, and doubtless this edition will find the same favor with students as did the former editions. All such books as this have undesirable influences in many ways, but seem to be essential from the student's standpoint, and no doubt they impart knowledge to some who would be too careless to read carefully and condense the matter of larger works. The illustrations are numerous, type clear, and the size convenient. J. P.

